# The sight of one’s own body: Could qEEG help predict the treatment response in anorexia nervosa?

**DOI:** 10.3389/fpsyg.2022.958501

**Published:** 2022-10-10

**Authors:** Marek Susta, Gustav Bizik, Anna Yamamotova, Svojmil Petranek, Marie Kadochova, Hana Papezova

**Affiliations:** ^1^Department of Public Health, St. Elisabeth University, Bratislava, Slovakia; ^2^Department of Psychiatry, Aalborg University Hospital, Aalborg, Denmark; ^3^Department of Physiology, Third Faculty of Medicine, Charles University, Prague, Czechia; ^4^Health Care Facility, Department of the Interior, Prague, Czechia; ^5^Department of Psychiatry, First Faculty of Medicine, Charles University and General University Hospital, Prague, Czechia

**Keywords:** anorexia nervosa, qEEG, treatment response, facial expressions, perception, disgust

## Abstract

**Aims of the study:**

The study aims to identify the differences in brain activity between participants with anorexia nervosa and healthy control using visual stimulus conditions combined with the quantitative dense-array EEG recording analysis method called Brain Activation Sequences (BAS).

**Materials and methods:**

23 participants with anorexia nervosa and 21 healthy controls were presented with visual stimuli, including the subject’s facial expressions and body images. The 128-channel EEG data were processed using BAS and displayed as activity in up to 66 brain regions. Subsequent cluster analysis was used to identify groups of participants exhibiting area-specific activation patterns.

**Results:**

Cluster analysis identified three distinct groups: one including all healthy controls (HC) and two consisting of all participants with anorexia (AN-I with 19 participants and AN-II with four participants). The AN-I and AN-II groups differed in their response to treatment. Comparisons of HC vs. AN confirmed the dominance of the right cerebral hemisphere in participants with anorexia nervosa in two of the three reported conditions. The facial expressions condition, specifically the facial reaction expressing disgust, indicates the existence of a social attentional bias toward faces, whereas emotions remained undetected in participants. High limbic activity, medial frontal gyrus involvement, low fusiform cortex activity, and milder visual cortex activity in healthy controls compared to participants indicate that the facial expression stimulus is perceived by healthy subjects primarily as an emotion, not as the face itself. In the body image condition, participants showed higher activity in the fusiform gyrus and right insula, indicating activation of the brain’s “fear network.”

**Conclusion:**

The study describes a specific pattern of brain activation in response to facial expression of disgust and body images that likely contributes to social-cognitive and behavioral impairments in anorexia. In addition, the substantial difference in the pattern of brain activation within the participants with AN and its association with treatment resistance deserves special attention because of its potential to develop a clinically useful prediction tool and identify potential targets for, for example, neuromodulatory treatments and/or individualized psychotherapy.

## Introduction

Despite the increasing emphasis on prevention, treatment, and research in this area, the global impact of anorexia nervosa (AN) does not appear to have diminished in recent decades. While the overall incidence appears relatively stable, there has been an increase in the high-risk group (15–25 years; Smink et al., 2012), mortality remains one of the highest among psychiatric conditions (Roux et al., 2013), the disorder is evolving into a chronic condition in a significant group of participants, and treatment outcomes have lagged behind expectations, particularly in terms of long-term effect and impact on psychiatric symptoms vs. weight (Murray et al., 2019). A better understanding of the neurobiology of this disorder leading to new diagnostic and treatment response prediction tools is therefore pressing.

One of the prominent characteristics of AN is an abnormal perception and evaluation of body shape, which is manifested mainly by an overestimation of the patient’s body size (Uher et al., 2005). Therapeutic approaches focus primarily on cognitive-behavioral therapy, supported by pharmacotherapy for common psychiatric comorbidities, especially anxiety and depression. According to current treatment models and strategies, the main goal is to achieve a change in emotional processing (Halls et al., 2021). However, the prevailing insight into the dynamics of these changes is deeply based on data obtained from measurements of body proportions, observation of patient behavior, and patient self-reports; methods for objective diagnosis and prediction of disease progression are still lacking (Seidel et al., 2018). This study attempts to address the problem described by Seidel regarding the current absence of methods to objectively diagnose and predict the development of AN by trying to answer the question of whether the brains of study participants produce patterns that distinguish between participants with AN and healthy controls when exposed to certain stimuli. The second question was whether there is a subgroup within the participants with AN with a specific response that reacts to the treatment differently or not at all.

One of the central irregularities described in AN is an increased sensitivity to punishment (Harrison et al., 2011). Research focusing on mindsets-shifting, central coherence, and decision-making disorders in participants with AN has shown that this is a stable feature even in recovered participants (Danner et al., 2012). In another experiment, participants with high levels of emotion dysregulation experienced an increase in AN symptomatology, whereas low levels of dysregulation predicted a decrease in symptom trajectory (Racine and Wildes, 2015). The authors of the revised cognitive-interpersonal model described AN-specific attention to detail and low willingness to change attitudes, which may have a heritable component. According to their findings, severely impaired global integration results in poor central coherence. Impaired social–emotional processing, manifested as attentional biases toward critical facial expressions and errors in interpreting and regulating emotions may be partly attributable to starvation.

A considerable body of work on AN has described the processing of visual stimuli, including various types of aversive images, such as emotional faces (Pollatos et al., 2008). Others have focused on the brain’s response to shape/weight and neutral words (Blechert et al., 2011) as measured by event-related EEG potentials (ERPs) or functional imaging methods (Frank and Kaye, 2012; Wright et al., 2015). A widely cited study by Uher et al. (2005) suggests that the tendency to display diagnosis-specific AN symptoms (e.g., desire for thinness and fear of fatness) and behaviors (restriction, purging) is conceivable as preferential activation of specific neural pathways and circuits. However, according to a detailed review by Fuglset et al. (2016), many fMRI studies have focused on body and appearance, emotions, and other aspects of AN, including the identification of different brain regions involved in the processing of visual stimuli, with mixed results. For example, some studies have found a bias towards facial expressions (Harrison et al., 2010), others have found a bias away from faces (Fassino et al., 2004), while some have found no bias at all (Castro et al., 2010). All these studies focus on dynamic results in the form of ERPs with low spatial accuracy or fMRI experiments lacking temporal resolution. Neural visual stimulus processing in brain sub-networks can be of transient and fast nature. The fMRI might not have detected certain regions because of its ability to capture brain activation over more extended periods (Trautmann et al., 2009). Other brain areas involved in processing the stimulus, either emotional or cognitive, could stay invisible to the approach (Im, 2007).

We have applied two types of visual stimuli activating brain pathways to investigate differences in stimulus processing between participants and healthy controls using high-density nonlinear self-learning qEEG analysis. For the purpose of completeness, we would like to note that the evaluation of classical EEG is based on visual assessment of fit between the displayed signal and predefined patterns, whereas qEEG is based on mathematical signal processing and, strictly speaking, does not require visual assessment. The design of our study is based on the findings from experiments cited in the previous paragraphs, but seeks to minimize some of their technological limitations by combining high-density EEG and signal source localization. The experiment results were expected to confirm differences in gyrus usage between participants and controls, and to assess to what extend the group of participants with AN is homogenous.

The study design included tasks to confirm the validity of the obtained results. These included the repeatedly published dominance of the right hemisphere in AN; differences in stimulus processing between participants with AN and healthy controls in gyrus involvement supporting the existence of emotional and attentional biases in face processing and body image mismanagement in participants; specifically, in the facial expression of disgust, repeatedly pointed out by other researchers as eliciting a specific response (Kucharska-Pietura et al., 2004; Trautmann-Lengsfeld et al., 2013). The Brain Activation Sequences (BAS) method was chosen for its temporal resolution and ability to identify a sequential set of brain sites used in stimulus processing. The relevant section also presents a more conventional way of displaying the results.

## Materials and methods

### Study participants

Twenty-three female participants with a diagnosis of anorexia nervosa (AN), hospitalized in the Eating Disorder Treatment Unit (EDTU) at the Psychiatric Clinic of the 1st Faculty of Medicine, Charles University in Prague participated in the experiment. All participants met the criteria for the restrictive type of AN according to the DSM-5 (American Psychiatric Association. Dsm-5 Task Force, 2013), and all diagnoses were independently confirmed by two psychiatrists from the EDTU. The majority (82.6%) of participants were on medication including selective serotonin reuptake inhibitors, benzodiazepines, noradrenergic and specific serotonergic antidepressants, atypical antipsychotics, and serotonin antagonists and reuptake inhibitors. The control group consisted of 21 healthy volunteers, recruited mainly from female students, similar in age and education to the participants with AN. All students who formed the healthy control group expressed interest in joining the experiment during the lectures in which the design of the experiment was presented. They were not financially rewarded for their participation, but motivated by their interest in the subject. All healthy controls passed the Eating Disorder Examination Questionnaire (EDE-Q) screening (Aardoom et al., 2012) and psychiatric examination for all forms of eating disorders with negative results. Descriptive statistics for both groups are shown in Table 1. Since the sample did not show a normal distribution, we present descriptive statistics in a format that allows a more detailed description of the population. None of the cases were the participants in the initial phase of treatment; all of them were past the halfway point in terms of planned hospitalization time. Status of participants with AN was continuously updated and during the experiment data processing phase, all these participants had completed their hospitalization so it was possible to evaluate the treatment outcome. Treatment outcome was rated by the attending psychiatrists on a scale of −2 to +2. Zero indicated no treatment effect, negative values deterioration, positive values improvement to significant improvement in the case of the outcome coded as +2.

**Table 1:** Descriptive statistics of study participants.

Category	Parameter	Min	Max	Median	Inter-quartile range	95% CIs	
						Lower bound	Upper bound
AN; *N* = 23	Age (years)	18	44	22	9	21.3	26.8
	Height (cm)	161	187	167	8	165.2	170.5
	Admission BMI	9.7	17.24	14	2.7	13	14.7
	Discharge BMI	12.6	18.7	16.1	2.6	15.7	17.1
HC; *N* = 21	Age (years)	18	35	23	5.5	22.4	26.4
	Height (cm)	159	185	172	12.5	168.6	175
	Examination BMI	18.69	24.8	20.8	1.39	20.4	21.7

BMI, Body Mass Index.

### Data acquisition

The basis of the project was the examination of data obtained from qEEG recordings, so we present the parameters associated with the technique and the chosen procedures. The recording was performed in a room designed for recording biological signals, shielded by a Faraday cage. A 128-channel EGI Net Amps 300 EEG amplifier, combined with a Geodesic Sensor Net, was used for recording, utilizing sponges moistened with a weak aqueous solution of potassium chloride for contact with the scalp. All participants were seated in a comfortable chair and fitted with a HydroCel Geodesic Sensor Net with 128 electrodes whose impedance was kept below 50 kΩ throughout the experiment to ensure maximum recording quality (Ferree et al., 2001). All channels used the reference electrode Cz for acquisition. A band-pass filter with cutoff frequencies set from 0.1 to 100 Hz with 3 dB attenuation, a gain of 1,000, and a sampling frequency of 500 Hz was used for recording, and a 16-bit A/D converter was used for digitizing the analog signal.

#### Stimulus presentation

**Figure 1:**
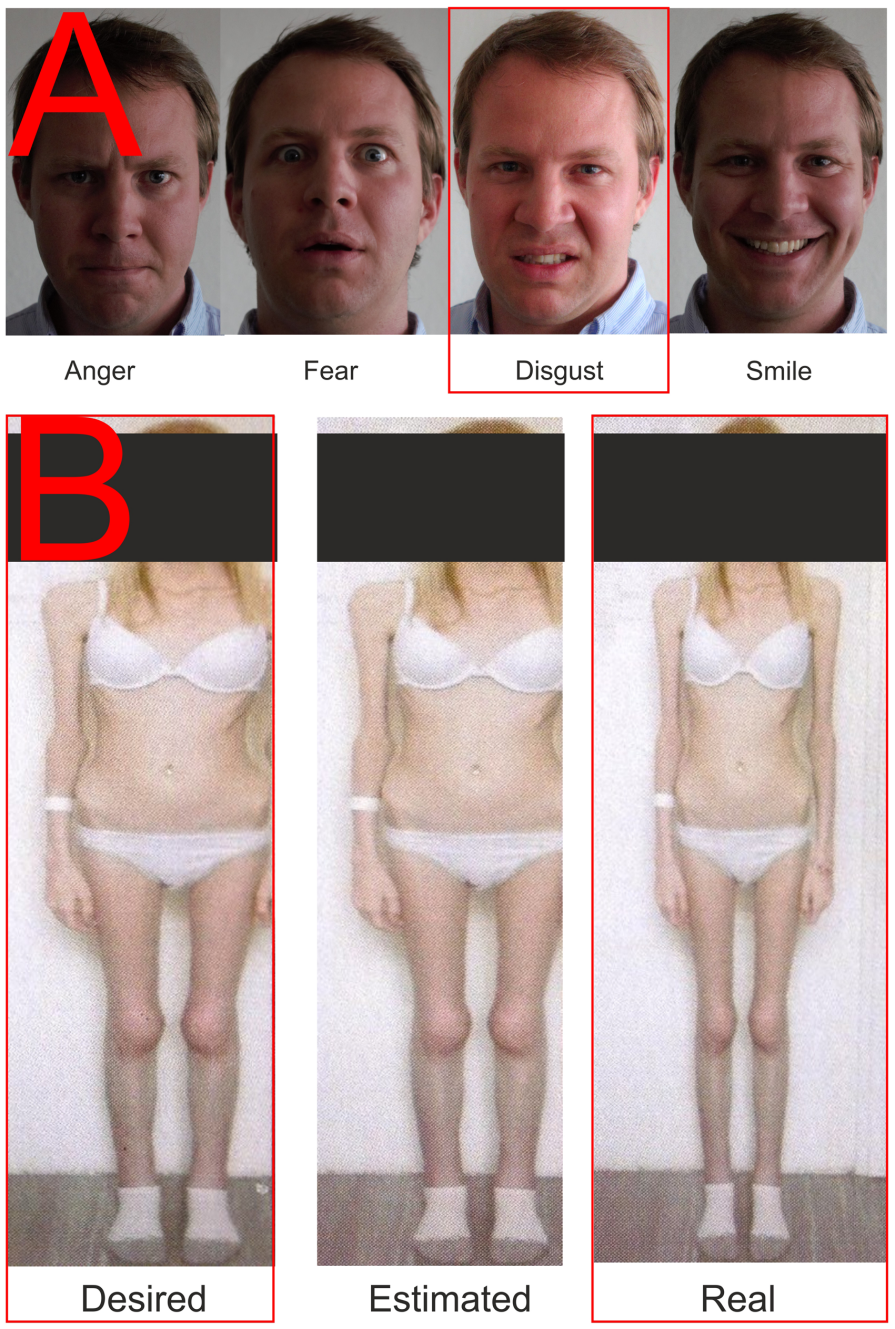
Examples of presented stimuli. **(A)** Facial expressions; **(B)** Body images. The red rectangle highlights the stimuli whose results are described in this paper.

Visual stimuli were presented to all participants in four blocks of 50 stimuli each. All pictures of a given set were displayed randomly on the screen, but the probability of displaying each picture was uniform and the display time was set to 1 s to cover the potentials associated with emotion and cognitive stimulus processing (Trautmann-Lengsfeld et al., 2013). In addition, participants were provided with a keypad in their dominant hand to express their feelings about the stimulus, in other words, whether they liked or disliked the presented image. Although manual controls during EEG recording increase the risk of motion artifacts, we wanted the presence of buttons to ensure that the participant was paying maximum attention to the stimuli. In addition, the presentation screen was equipped with a camera synchronized with the EEG recording to check the participant’s behavior to see if they were watching the screen and engaged in other activities. During the EEG recording, participants were only asked to watch the screen and respond to the images by pressing a like-dislike buttons. The EEG was recorded for all stimuli shown in Figure 1, only those marked with a red border in the figure are presented in this paper because the volume of data presented seems to be sufficient to evaluate the tested hypotheses.

##### Facial expressions

A total of four pictures were used in the condition focusing on the emotional response elicited by the presentation of faces with a particular emotional expression. Three of them expressed negative emotions (fear, anger, and disgust) and one positive one, containing a smiling face. The set used is shown in Figure 1A. These facial expressions have been used since 2010 and were pre-tested on the group of *N* = 104 using the E-prime software application with a four-button response box (each button marked with one emotion). Each expression was presented to the subject 25 times in random order; there were up to 2 s to respond. Average consistency responses (mean proportion consistent answers) were Anger = 94.16%, Fear = 84.46%, Disgust = 82.65%, and Smile = 96.27%. The male face was selected based on the findings of Adolph and Alpers (2010): “…more intense male expressions may be more potent in inducing emotional responses.” The consistency in our set was generally higher than those of a large and detailed study performed by Tottenham et al. (2009).

##### Stimuli from the anamorphic software

With the consent of all participants, standardized photographs of their bodies in their underwear were obtained by trained medical staff and then uploaded to Anamorphic software (Symplex Information Solutions, United Kingdom). This software can identify and adjust the body’s width in the embedded photograph. Next, participants were asked to use a mouse slider to create the body they would like to have (desired body) by modifying the original image, and then the body they thought matched what they actually looked like (perceived body). Finally, the two newly created and the original served as stimuli in a second condition, as shown in Figure 1B. Anamorphic software has been used for a long time in the department. We were not pre-testing these pictures to prevent priming or habituation. Each set was created for a particular participant based on images of her own body and her settings of the distorted (desired and estimated) versions. The pictures were then destroyed except for the one presented in Figure 1 (with the participant’s permission). Participants first modified their photographs in the Anamorphic software, which were then entered into the stimulus presentation software by the research team. No EEG recording was performed while the photographs were being edited.

### Data processing

Continuous EEG recording was filtered using a 30 Hz low-pass filter and a 0.3 Hz high-pass filter and divided into epochs according to stimulus type-100 ms from the stimulus presentation mark +1,000 ms. Next, epochs contaminated by eye or motion artifacts were eliminated, and individual bad channels were replaced segment by segment by spherical spline interpolation. Segments that passed all the above steps were averaged. Finally, averaged segments were re-referenced with a Polar Average Reference Effect (PARE) correction to estimate the zero integral of the surface potential (Junghofer et al., 1999) and adjusted to a baseline of 100 ms before the stimulus presentation marker.

#### Checking the meaningfulness of the measured data

**Figure 2:**
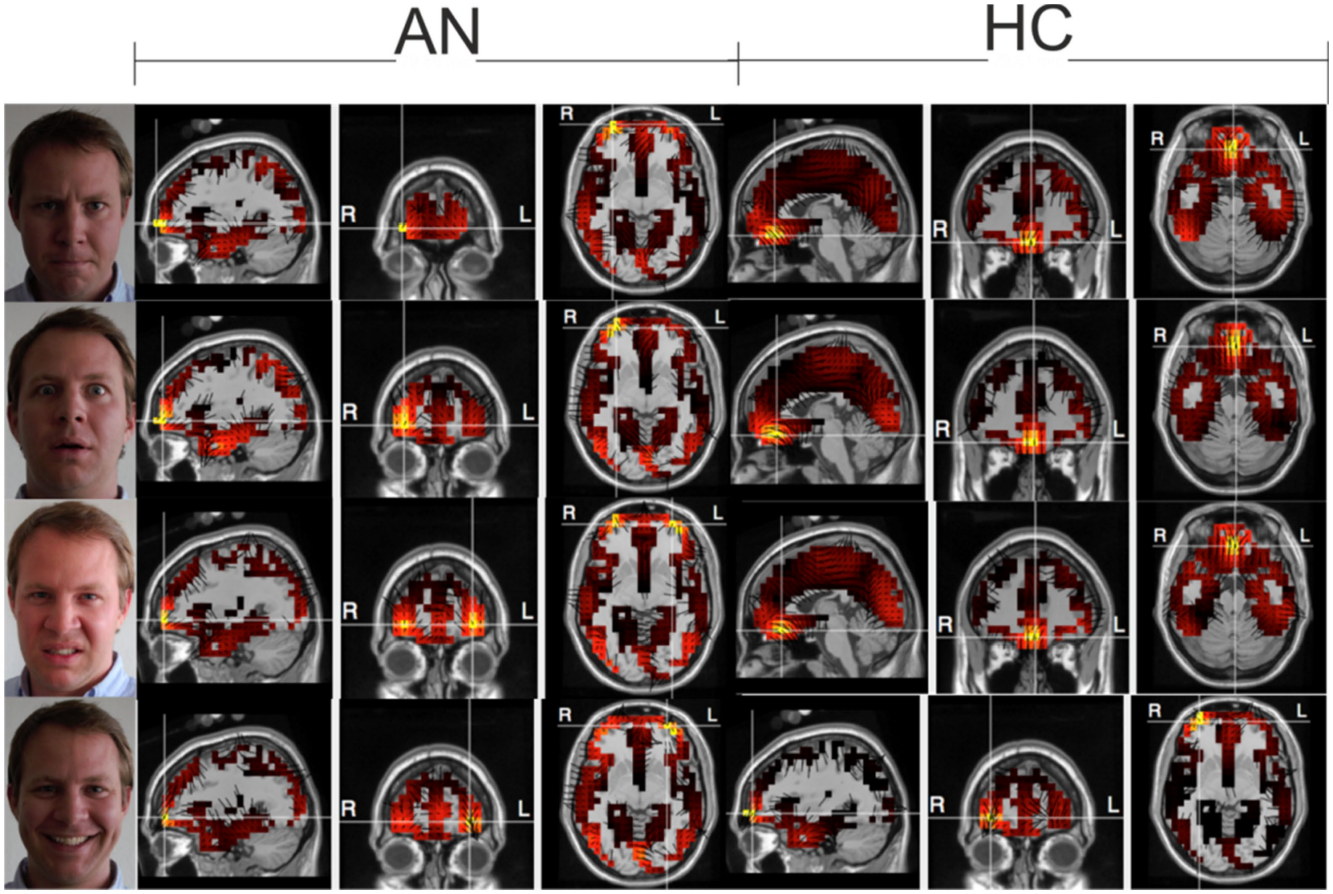
Electrical sources at the selected time as a check of the consistency of the measured data. Top-down Anger-Fear-Disgust-Smile, during maximal activation using the LAURA method. 2,400 voxels representing gray matter in the Montreal Neurological Institute average MRI (2,394 plus six thalamic sources) are registered in Tailarach space with a typical MRI image of the subject. Three orthogonal dipole moments are calculated for each voxel with linear inverse estimation. The color palette, which is kept constant for this series of images, reflects the effective value of the three dipole moments of each voxel.

Before performing the actual BAS processing, we wanted to make sure that the data obtained would have properties consistent with results presented in published studies using other technologies. Therefore, for verification, we computed both classical evoked potentials (not shown, they have been successfully published many times by other research teams), and further processed the data for display in fMRI format as shown in Figure 2. The results of these two ancillary computations were then compared with the results of previously published experiments on the same topic. The comparison results are discussed in more detail in the first part of the Discussion paragraph.

#### Data processing with the BAS method

The basis of the method is the calculation of the signal source from qEEG data. Sufficient accuracy of the digitized data is ensured in EEG recording by applying Nyquist’s theorem, which determines the necessary sampling frequency depending on the frequency of the processed signal (Nyquist, 1928). The highest frequency recorded fell in the β-2 band, and the 500 Hz sampling rate used thus exceeded the minimum required by the theorem several times over. Similarly, in terms of the needs of the signal source algorithm, the spatial “Nyquist,” specifying the minimum number of leads for qEEG recording, can be formulated. The minimum number of leads for brain gyrus-level computations reported in the literature is 60 (Srinivasan et al., 1998). The 128-channel technology used is twice the required minimum. The algorithm described by De Peralta et al. (2001) was used to compute the signal source based on the local autoregressive average (LAURA) calculation. The reason for choosing this particular algorithm is its output; LAURA does not focus only on the most intense source; it can calculate multiple signal sources at a given time (De Peralta-Menendez and Gonzalez-Andino, 2002). In the next step, BAS determines from all local autoregressive average data of all study participants a state space according to which it constructs and creates a system of differential equations expressing the dynamics of gyral activity. Each subject’s data from the first stage of the computation (the signal source calculation) are then computed by the system of equations and the output is a sequence of gyri active at the time of stimulus processing. The output table contains only the record code; it does not indicate which group the output belongs to (AN, healthy controls). Thus, the software does not know whether it currently processes data from a participant with AN or HC. The BAS output table, which contains no information about group membership and only tells about the activity of individual brain gyri during stimulus processing, can be used as input for cluster analysis. The goal of the whole operation is to see if the cluster analysis partitions the unordered BAS output table according to group membership or, stated differently, whether it forms two clusters, one occupied by AN and the other by healthy controls.

A detailed description of the method was published in Susta et al. (2015), and the application of the technique to hypersomnias of central origin was described in Susta et al. (2016).

## Results

**Figure 3:**
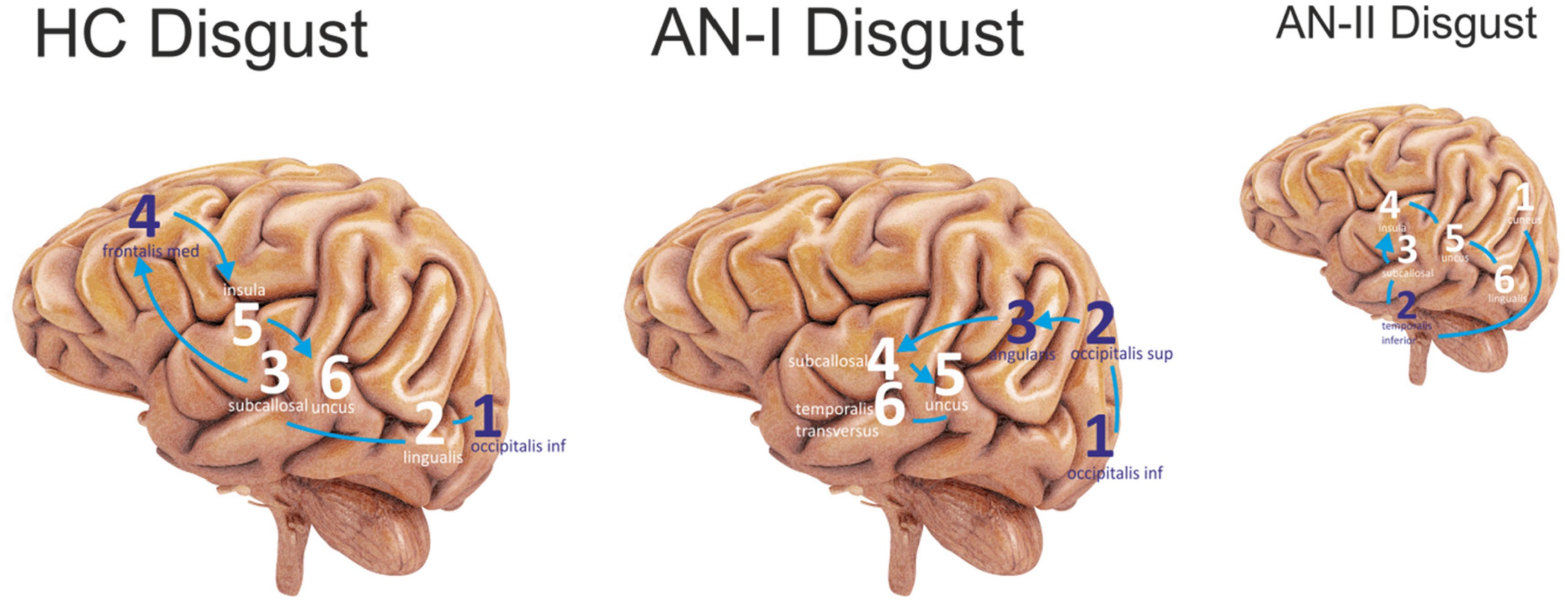
The first six sites that were most active in the Disgust condition were sequenced. The purple color represents the lateral sites, and the white color refers to the medial areas. Connecting lines without arrows show the same level of activity. HC Disgust refers to the HC group, AN-I Disgust refers to the AN-I group, and the smaller AN-II Disgust image refers to the AN-II group in results (Table 2).

**Figure 4:**
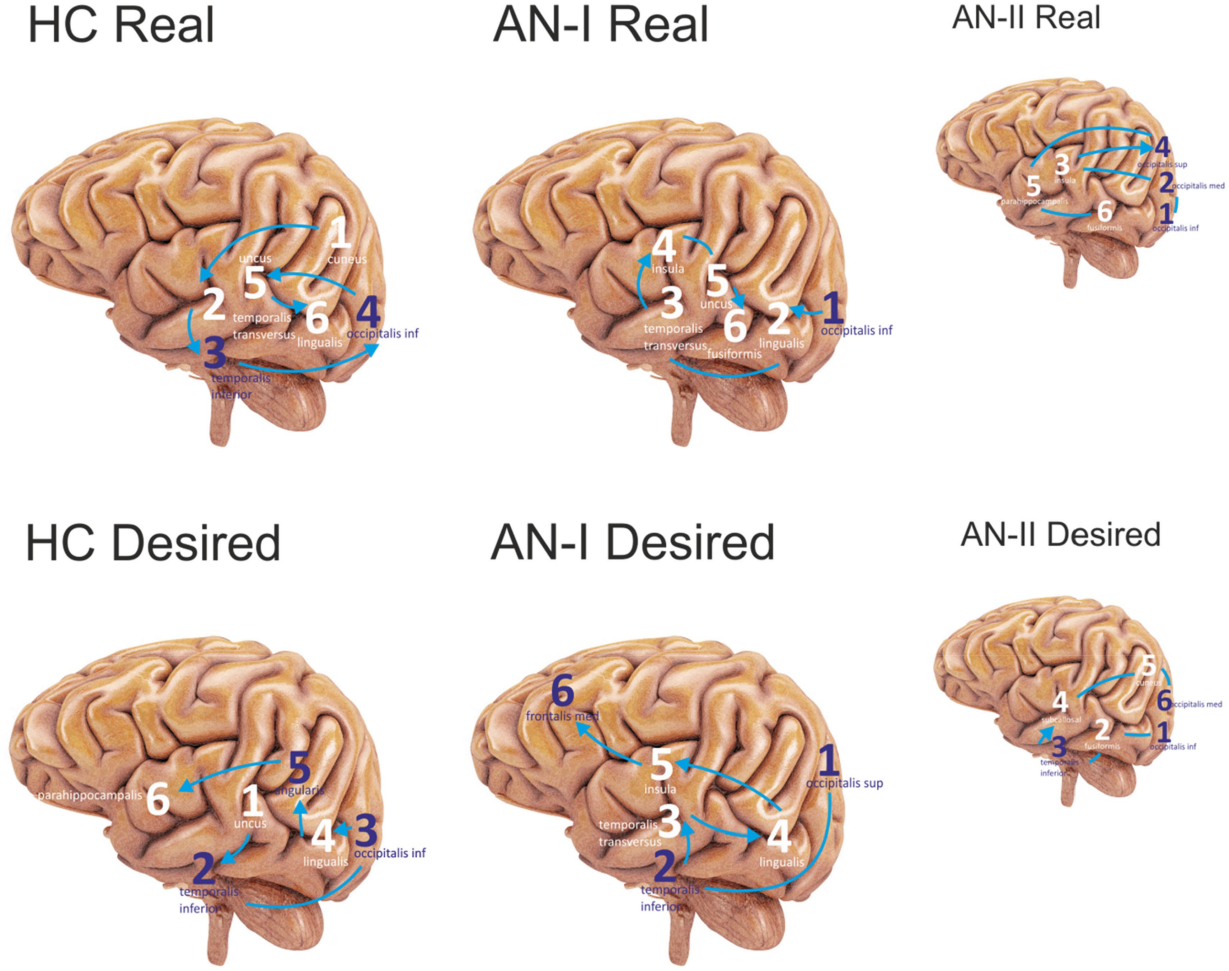
Sequenced the first six most active sites in the Anamorphic condition. Purple numbers represent lateral sites; white refers to medial sites. Connecting lines without arrows show the same level of activity at a given time. The HC corresponds to group HC, AN-I refers to group AN-I, and the smaller figure AN-II refers to group AN-II in Table 2. The upper group of figures describes the activity elicited by the Real body image, and the lower group the response to the distorted, Desired body shape.

Brain activation sequences provide two types of outputs. The first was described in the previous paragraph. It is a table illustrating the gyral activity of each study participant, and the second is a graphical representation of the same. The graphical representation, the output of which is shown in Figures 3, 4, is only for a cursory visual comparison of the measured values.

The original assumption that cluster analysis would form two clusters, one containing the outcomes of participants with AN and the other of healthy controls, was not confirmed. Instead, the output of the K-cluster analysis was three clusters, the first including only HCs, and the second and third containing study participants with AN.

As noted, the BAS output is a table with binary data on gyral activity during stimulus processing. After grouping them according to the cluster analysis results, it was possible to statistically evaluate the differences between the clusters. As this is a comparison of binary parameters, chi-square was used. Due to the high number of parameters being compared, there was a risk of a Type-I error, and a post-hoc Bonferroni correction was applied.

Table 2 shows the complete statistics of all results discussed in this paper. We deliberately do not report results for all stimuli and all combinations for two reasons. First, the extent of the results presented in detail, in our opinion, answers the research questions, and second, showing all the data would impose an unsustainable demand on the size and number of output elements. The Real and Desired columns refer to the Anamorphic condition, while the Disgust column contains the results of the Emotional Faces condition. The sub-columns labeled Final Cluster Centers contain the results of the cluster computation, and the adjacent column displays the Pearson chi-square and the exact two-sided sigma (*p*). The bottom two rows describe activity totals for the left and right hemispheres to answer the secondary question of whether the frequently reported right-sided dominance in AN is confirmed in the presented condition. Right hemisphere dominance was found in AN for the desired distorted body image and the emotional face expressing disgust, but not for the actual body image, where the right hemisphere was also dominant in HC.

**Table 2:** Numerical results of the BAS outputs cluster analysis (Final Cluster Centers sub-columns) and Pearson chi-square (Pearson sub-columns).

Location	Real					Desired					Disgust				
	Final Cluster Centers			Pearson’s		Final Cluster Centers			Pearson’s		Final Cluster Centers			Pearson’s	
	AN-I	AN-II	HC	Chi-square	*p*	AN-I	AN-II	HC	Chi-square	*p*	AN-I	AN-II	HC	Chi-square	*p*
Occipitalis Inferior L	0.00	1.00	0.00	44.00	0.000	-	-	-	-	-	0.05	0.00	0.90	32.88	0.000
Occipitalis Inferior R	1.00	0.00	0.86	24.77	0.000	0.00	1.00	0.90	36.74	0.000	0.95	0.00	0.05	36.25	0.000
Occipitalis Medius L	0.00	1.00	0.14	24.77	0.000	0.00	0.75	0.05	23.40	0.001	-	-	-	-	-
Occipitalis Superior R	0.00	0.75	0.00	32.19	0.000	0.95	0.00	0.00	40.08	0.000	0.95	0.25	0.05	33.31	0.000
Uncus L	0.84	0.00	0.86	14.97	0.002	0.79	0.00	1.00	22.77	0.000	0.74	0.75	0.76	0.03	1.000
Lingualis L	0.89	0.25	0.71	7.59	0.019	0.05	0.00	0.43	7.02	0.030	0.00	0.75	0.90	33.76	0.000
Lingualis R	0.00	0.00	0.05	1.21	1.000	0.89	0.00	0.76	14.13	0.001	0.00	0.00	0.05	1.12	1.000
Subcallosal L	0.00	0.00	0.14	3.52	0.307	0.05	0.00	0.33	6.26	0.044	0.11	0.00	0.90	29.57	0.000
Subcallosal R	-	-	-	-	-	0.00	0.75	0.05	23.40	0.001	0.79	1.00	0.05	27.42	0.000
Cuneus R	0.21	0.00	1.00	31.12	0.000	0.11	0.75	0.38	8.01	0.018	0.05	1.00	0.10	23.39	0.000
Angularis L	0.05	0.25	0.14	1.61	0.471	0.25	0.05	0.71	37.17	0.000	0.89	0.00	0.00	36.45	0.000
Angularis R	0.00	0.00	0.05	1.21	1.000	-	-	-	-	-	0.00	0.00	0.05	1.12	1.000
Fusiformis L	0.37	0.75	0.19	5.23	0.070	0.00	0.05	0.00	1.34	0.523	0.50	0.42	0.05	8.88	0.011
Fusiformis R	0.42	0.25	0.10	5.65	0.070	0.05	1.00	0.00	34.59	0.000	0.53	0.25	0.05	8.95	0.012
Insula L	0.21	1.00	0.14	13.44	0.002	0.11	0.25	0.05	1.74	0.432	0.05	0.25	0.43	7.51	0.022
Insula R	0.84	0.25	0.24	18.55	0.000	0.84	0.00	0.05	29.32	0.000	0.00	0.75	0.81	27.19	0.000
Temporalis Inferior L	0.53	0.00	0.62	5.16	0.093	0.95	0.00	0.90	23.39	0.000	-	-	-	-	-
Temporalis Inferior R	0.00	0.00	0.90	36.62	0.000	0.05	1.00	0.05	27.86	0.000	0.00	1.00	0.00	44.00	0.000
Temporalis Transversus L	-	-	-	-	-	0.89	0.00	0.00	36.45	0.000	0.74	0.25	0.05	33.31	0.000
Temporalis Transversus R	0.89	0.00	0.95	23.50	0.000	0.21	0.00	0.14	1.17	0.607	0.53	0.50	0.10	2.93	0.257
Cingularis Posterior R	0.16	0.00	0.05	1.91	0.326	-	-	-	-	-	0.05	0.00	0.05	0.22	1.000
Parahippocampalis R	0.00	0.75	0.05	23.40	0.001	0.32	0.00	0.62	20.20	0.000	0.00	0.00	0.10	2.29	0.578
Frontalis Medius L	-	-	-	-	-	0.37	0.50	0.19	2.40	0.340	0.00	0.00	0.86	33.36	0.000
LH activity	2.90	4.25	2.95			3.09	1.10	3.47			3.08	2.42	4.86		
RH activity	3.52	2.00	4.24			3.80	5.00	3.14			3.85	4.75	1.43		

The bottom two rows contain the sum of left and right hemisphere gyri. AN-I *N* = 19, AN-II *N* = 4, and HC *N* = 21.

For the disgust-expressing face condition, the results in Table 2 (AN-I vs. HC) show statistically significant differences, namely in the gyrus occipitalis inferior (left, right), occipitalis superior (right), lingualis (left), subcallosal (left, right), angularis (left), insula (left, right), temporalis transversus (left), fusiform gyrus (left, right), and frontalis medius (left). The first six most active locations are shown in the sequence in Figure 3.

The results of the condition with the participant’s own body image are presented in the Real column of Table 2 (AN-I vs. HC) and show statistically significant differences between groups in response to the real body image in the cuneus (left), fusiform gyrus (right), insula (right), and temporalis inferior (right).

The results in the Desired column in Table 2 (AN-I vs. HC) show statistically significant differences between groups in the occipitalis superior (right), lingualis (left), insula (right), and temporalis transversus (left) in response to the distorted, supposedly desirable image of the body proper. Figure 4 then shows the gyrus sequences for both Real and Desired.

However, we consider dividing the participants’ results into two clusters to be a result worthy of special attention. Anamnesthetically, no difference is evident between the members of the two clusters. This became apparent when the treatment outcome data were obtained. In the entire cohort of all 23 individuals with AN, there were only four who deteriorated during hospitalization. These four formed a separate cluster, designated as AN-II in the table. A *post-hoc* analysis showed substantial statistically significant differences between AN-I and AN-II across all three conditions. For the disgust-expressing face condition, both groups differed in occipital superior (right), occipital inferior (right), cuneus (right), angularis (left), insula (right), temporalis inferior (right), and lingualis (left). For the real body image condition, both groups differed in occipitalis superior (right), occipitalis medius (left), occipitalis inferior (right and left), uncus (left), temporalis transversus (right), insula (left), and parahippocampalis (right and left). For the real body image condition, both groups differed in occipitalis superior (right), occipitalis medius (right), occipitalis inferior (right and left), insula (right and left), lingualis (left), uncus (left), and parahippocampalis (right and left). For the desired body image condition, both groups differed in occipitalis superior (right), occipitalis medius (right), occipitalis inferior (right), uncus (left), cuneus (right), insula (right), temporalis inferior (right and left), temporalis transversus (left), supramarginalis (right), subcallosal (right), and fusiformis (right).

## Discussion

Considering the results of the analyses carried out, the discussion focuses on three main themes: (1) disgust condition, (2) anamorphic condition will compare specifically AN-I group and HC group because of statistical robustness, and (3) findings related to the relatively small but clinically extremely interesting AN-II group.

Results from the Disgust stimulus condition confirm the right hemisphere (RH) dominance reported by numerous research groups (Grunwald et al., 2004; Galusca et al., 2008; Trautmann-Lengsfeld et al., 2013). The source localization algorithm does not involve subcortical structures; the activity of the limbic system is accessible only indirectly through sites with solid connections to its prominent parts. Insula integrates projections from the lateral and basolateral amygdala (Gogolla, 2017). High visual cortex and low limbic system activity, together with increased fusiform gyrus activity in AN, confirm conclusions published by Harrison et al. (2010) and Ashwin et al. (2006), suggesting the existence of social attentional bias toward faces while the emotion remains undetected. Interestingly, this finding is in line with the observed impairments in emotion recognition and avoidance of emotional faces in non-clinical population with high scores on Eating Disorder Inventory (Sharpe et al., 2016).

Insula is extensively mentioned in papers as a site highly active during processing disgust (Jabbi et al., 2008; Verstaen et al., 2016). High insular activity, the involvement of the medial frontal gyrus, low fusiform gyrus activity, and more moderate visual cortex activity in HC compared to AN indicate that the stimulus is perceived by healthy participants primarily as an emotion, not as a face. From a psychological perspective, these findings are in line with the observations associating low levels of mentalization and the ability to understand another person’s perspective with the development of AN (Rothschild-Yakar et al., 2010).

Moreover, it is worth to note a substantial overlap (75%) between the active regions reported in Table 2 and brain circuitry identified by Sato et al. (2019), who studied individuals with Autism Spectrum Disorders using dynamic facial expressions. A morphometric experiment by Bjornsdotter et al. (2018) discovered lower temporal grey matter volumes in ASD. Our findings in the AN-II group show low activity in temporal regions that could be explained by the presence of ASD traits.That might help to explain the differences in interactions between limbic and cortical structures in AN and HC discussed above.

The Anamorphic condition with real and distorted desired body images confirmed RH dominance for the desired stimulus but not for the actual image of the subject’s body. Reaction to the real image in AN-I and HC differs mainly in limbic system activity, but both groups utilize brain regions connected to anxiety and fear.

Participants with AN show elevated activity in the fusiform gyrus and right insula, confirming the findings of Seeger et al. (2002), indicating an activation of the brain’s “fear network.” Similar, but a weaker reaction to the real body stimulus in HC, including fusiform gyrus, right parietal areas, and left cingulate with limbic system activity correlates with anxiety generated by the task of judging a person’s own body. Even in healthy women, body image and anxiety mechanisms are implicated in these tasks (Miyake et al., 2010; Frank and Kaye, 2012).

Response to the desired image differs from the real stimulus in fusiform gyrus, parietal and limbic system activity. The aforementioned study by Seeger et al. (2002) also found high activity in the amygdala and fusiform gyrus in response to distorted body images. In contrast, a subsequent study with the same design found activation not in the amygdala but in frontal and parietal regions thought to be involved in integrating body schemas and body ownership (Wagner et al., 2003).

Our study discovered activity in the limbic system and the frontal and parietal regions. That, together with inactive fusiform gyrus, might suggest a pending process of body evaluation accompanied by an emotional response that differs between AN-I and HC groups (Paslakis et al., 2021). The reaction to the image in AN-I, similar to defective pain sensation, confirms erroneous own body perception associated with anxiety and fear that often leads to self-hatred and various forms of auto-destructive behavior as a result of malnutrition, lack of self-acceptance, and traumatization (Cucchi et al., 2016; Yamamotova et al., 2017). The aversion reaction detected in the participants from the AN-I group in response to the “desired” image of their bodies (Figure 1B) may have arisen as a result of the therapeutic interventions from which the participants concluded that this is how they should look, however, their internal beliefs were still in conflict with an image significantly broader than the reality. In the healthy controls, the “desired” image was always thinner than reality, but the data showed that the discrepancy between perceived reality and the “desired” shape did not evoke any significant emotion. The “desired” body image was subsequently commented on by some with the words: “It would be nice, but I think I look good already.”

Differences in utilization of the parahippocampal gyrus addressed the study of Shirao et al. (2003), who found its negative relation to the Eating Disorders Inventory-2 score.

The attempt to understand and possibly interpret the distinct differences between the two cluster in participants with anorexia is necessarily limited at the current stage of our knowledge because of the following factors: (1) cluster analysis is performed on a complex dynamics system which by itself make any traditional (mechanistic) analogies inappropriate, (2) no EEG studies are using the same protocols (to the best of our knowledge), leaving us with the options of indirect comparisons with fMRI literature, however (3) without any studies focusing on differences of functional pattern in a group of participants with AN from the treatment response perspective.

The list of the most active regions during real-body and desired-body conditions is consistent with the findings of the review article by Esposito et al. (2018), which shows that fMRI studies using body image perception and body size estimation tasks suggest modifications in the activity of the extrastriatal body region, fusiform body region, and parietal cortex. However, as with the fMRI studies reporting that the distinction between anorexic participants and controls was not clear, our findings did not allow us to follow up on the conclusion of differences between AN-I and AN-II when considering specific regions, including the superior occipital area, the angular gyrus, and the fusiform gyrus.

The pattern of activation in the desired-body condition found in both groups is consistent with Suchans’ finding (Suchan et al., 2013) describing a correlation between right-sided fusiform activation and incorrect body size, suggesting that one of the differences between AN-I and AN-II could be related to the more pronounced dysfunction in the treatment-resistant group.

In addition, the differential activation of the gyrus lingualis and cuneus between groups in all three conditions may further suggest differences in attentional control and impairment from self-referential processing, as these structures are involved in attention and avoidance coping in anorexia fMRI studies by Noda and Isobe (2021).

Regarding the disgust condition, the two groups differed mainly in the predominant activation of the insula, temporal region, and cuneus in AN-II, whereas none of these areas were active in AN-I. This pattern of activity is consistent with the accentuated response to the disgust cue in the treatment-resistant group, allowing us to hypothesize that if self-disgust does not change during AN treatment, residual levels of self-disgust could contribute to treatment resistance and make individuals vulnerable to relapse (Glashouwer and de Jong, 2021).

Finally, it seems interesting to note that although a functional study is lacking, a morphometric study by Favaro et al. (2015) found that participants with anorexia with poor 3-year treatment outcomes had significantly lower baseline gyrification compared to healthy controls and participants with full recovery at follow-up, even after controlling for the effects of disease duration, weight loss, and age of onset, supporting the idea of substantial differences in the neurobiology of treatment response suggested by our findings.

## Study limitations

We are aware that the number of participants may seem low to some. It should be noted that we tried to ensure as much homogeneity of data as possible, including identical therapeutic procedures, and so all participants admitted to the inpatient part of the AN unit at given time were included in the experiment. We wanted to ensure that any heterogeneity in outcomes was not attributable to the fact that some participants with AN had been treated by other psychiatrists or had participated in psychotherapy in another group or under the guidance of other therapists. Although discharge dates varied, all AN participants in the experiment were hospitalized and treated during the same period and thus interacted with the same medical and therapeutic personnel. It is clear that far-reaching generalizations cannot be made. On the other hand, AN-II group appeared to fully correlate with a treatment failure rate of 17.4%, reported by other specialized eating disorder treatment units.

The experimental results look promising, but the method used is new, and much more testing is needed to validate it in the ED. In addition, both samples were relatively small, and participants were medicated in many cases. This, together with the imperfection of the source localization method, the accuracy of the dynamic structure, and the unstable influence of the stimuli on different subjects, requires great caution before making a final judgment.

Drugs used to treat some participants are known to affect the EEG recording. Studies on the effect of antidepressants are rare, mostly available for sleep EEG, and their results are inconsistent. Most authors report a moderate effect on alpha and beta bands based on dosage or no effect (Banoczi, 2005; Dumont et al., 2005). In terms of the potential impact of medication, we could certainly consider involving only drug-naive participants or discontinuing medication for a necessary period of time, but we wanted to conduct the experiment in a realistic setting and not compromise the treatment process in any way. Therefore, we list the effect of medication as a potential limitation, but the degree of influence remains unknown.

A limitation of EEG as a primary data collection method is the known susceptibility to artifacts, which hinders the use of the design in restless subjects. Research groups equipped with qEEG amplifiers compatible with fMRI could substantially advance the search for a unified model of AN by obtaining spatially and temporally accurate data.

Further research is needed to show what exactly activation of specific regions means. The same question applies to the magnitude of inter-and intra-individual differences. Still, there is a chance that this or a similar method, considerably cheaper than fMRI or equivalent neuroimaging methods, could 1 day serve as a tool to predict treatment outcomes or to guide individualized treatment plans, perhaps as an alternative to fMRI-guided rTMS protocols for treatment-resistant anorexia.

## Data availability statement

The raw data supporting the conclusions of this article will be made available by the authors, without undue reservation.

## Ethics statement

The studies involving human participants were reviewed and approved by Ethics Committee of the First Faculty of Medicine, Charles University, Prague, CZ. The patients/participants provided their written informed consent to participate in this study. Written informed consent was obtained from the individual(s) for the publication of any potentially identifiable images or data included in this article.

## Author contributions

MS and GB: study design, acquisition, analysis, interpretation, and manuscript. AY and SP: study design, interpretation, and manuscript. MK: acquisition, analysis, and manuscript. HP: study design, analysis, interpretation, and manuscript. All authors contributed to the article and approved the submitted version.

## Funding

This research was supported by the project Q27/LF1, Cooperatio Program, research area Neuroscience and MH CZ–DRO VFN64165 and Proverbs grant NEURO2021-1.

## Conflict of interest

The authors declare that the research was conducted in the absence of any commercial or financial relationships that could be construed as a potential conflict of interest.

## Publisher’s note

All claims expressed in this article are solely those of the authors and do not necessarily represent those of their affiliated organizations, or those of the publisher, the editors and the reviewers. Any product that may be evaluated in this article, or claim that may be made by its manufacturer, is not guaranteed or endorsed by the publisher.
